# Recurrent spontaneous pneumothorax secondary to lung cystic lesions in a case of convalescent COVID-19: a case report and literature review

**DOI:** 10.1186/s12890-024-03169-5

**Published:** 2024-07-19

**Authors:** Yangzi Song, Jianmin Jin, Xuechen Wang, Jinguo Zhang, Zuojun Li

**Affiliations:** 1 Department of Infectious Disease, Beijing Tongren Hospital, Capital Medical University, No. 1, DongJiaoMinXiang, DongCheng District, Beijing, China; 2 Department of Respiratory and Critical Care Medicine, Beijing Tongren Hospital, Capital Medical University, No. 1, DongJiaoMinXiang, DongCheng District, Beijing, China

**Keywords:** COVID-19, Spontaneous pneumothorax, Cystic, Bulla, Pneumatocele

## Abstract

**Background:**

While spontaneous pneumothorax has been documented in COVID-19 patients, reports on recurrent spontaneous pneumothorax due to cystic lesions in convalescent COVID-19 patients are scarce. The progression of these lung cystic lesions remains inadequately explored.

**Case presentation and literature review:**

An 81-year-old male, a non-smoker with a history of rheumatoid arthritis, presented with fever, cough, and expectoration for 14 days. Initially diagnosed with moderate COVID-19, he deteriorated to severe COVID-19 despite adherence to local treatment guidelines. Successive identification of three cystic lesions termed “bulla” or “pneumatocele”, and one cystic lesion with air-fluid level, referred to as “pneumo-hamatocele” (PHC), occurred in his lungs. Gradual improvement followed anti-inflammatory therapy and optimal supportive care. However, on day 42, sudden worsening dyspnea prompted a computed tomography (CT) scan, confirming a right spontaneous pneumothorax and subcutaneous emphysema, likely due to PHC rupture. Discharge followed chest tube implementation for pneumothorax resolution. On day 116, he returned to the hospital with mild exertional dyspnea. Chest CT revealed recurrent right pneumothorax from a remaining cyst in the right lung. Apart from our patient, literature retrieval identified 22 COVID-19 patients with spontaneous pneumothorax due to cystic lesions, with a male predominance (95.6%; 22/23). Diagnosis of pneumothorax and lung cystic lesions occurred around day 29.5 (range: 18–35) and day 26.4 (± 9.8) since symptom onset, respectively. Except for one patient whose pneumothorax occurred on day seven of illness, all patients eventually recovered.

**Conclusions:**

Recurrent spontaneous pneumothorax secondary to lung cystic lesions may manifest in convalescent COVID-19 patients, particularly males with COVID-19 pneumonia. Chest CT around 2 to 3 weeks post-symptom onset may be prudent to detect cystic lesion development and anticipate spontaneous pneumothorax.

## Background

Common chest computed tomography (CT) findings in novel coronavirus disease 2019 (COVID-19) typically manifest as multiple patchy, ground-glass opacities progressing to or co-existing with bilateral consolidations across multiple lobes, often with peripheral distribution. While uncommon, CT scans have also revealed cystic lesions, such as bullae [1] or pneumatocele [2], in COVID-19 patients. However, the pathogenesis of these cystic lesions remains unclear, and their evolution is not fully elucidated. Although spontaneous pneumothorax has been documented in COVID-19 patients, reports of recurrent spontaneous pneumothorax secondary to cystic lesions are rare. In this study, we present a case illustrating the evolution of lung cystic lesions over four months, including recurrent spontaneous pneumothorax secondary to these lesions even three months after COVID-19 onset, with the aim of enhancing understanding of the disease, particularly the impact of cystic sequelae on convalescent patients. Additionally, we conducted a literature review and summary of spontaneous pneumothorax secondary to cystic lesions in COVID-19 patients.

## Case presentation

**Fig. 1:**
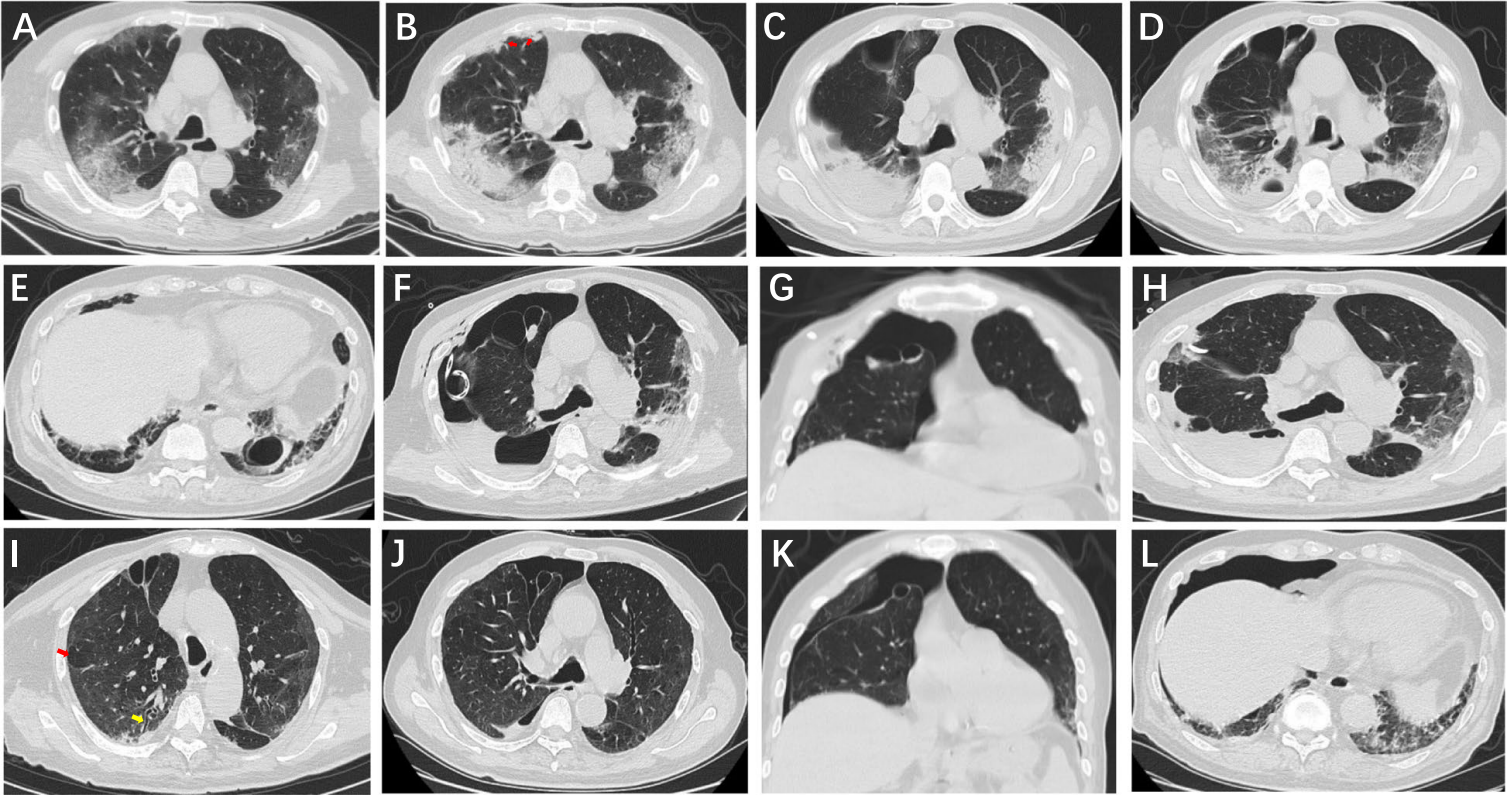
Evolution of lung lesions and secondary pneumothorax in a convalescent COVID-19 case. **A** Day 14: peripheral distributed ground-glass opacities (GGO) and patchy consolidation in both lungs. **B** Day 20: progression of lung lesions, more significant lung consolidation, along with subpleural air bronchogram in the right upper lobe (red arrow). **C** Day 26: in addition to aggravated lung consolidation, subpleural cystic lesions and the proximal bronchi with thickened walls and constricted lumen in the anterior segment of the right upper lobe. **D** Day 38: a cavity-like lesion with air-fluid level in the dorsal segment of the right lower lobe, and improved consolidation throughout both lungs. **E** Day 38: a cystic lesion in the posterior basal segment of the left lower lobe. **F** Day 42: evolution of the cavity-like lesion into an enlarged cystic lesion in the dorsal segment of the right lung, along with right pneumothorax and subcutaneous emphysema, while the two cystic lesions in the right upper lobe remained unaltered (transverse section). **G** Day 42: right pneumothorax and subcutaneous emphysema, with two cystic lesions in the right upper lobe (Coronal section). **H** Day 50: implementation of chest tube drainage produced amelioration of right pneumothorax and resolution of subcutaneous emphysema. **I** Day 80: consolidation of both lungs further improved, with subpleural cystic lesions constricted but still present, along with emphysema or air trapping (red arrow), and bronchiectasis (yellow arrow). **J** Day 116: recurrent right pneumothorax due to cyst rupture in the right upper lobe, amelioration of PHC in the right lower lobe, and significant absorption of lung consolidation (transverse section). **K** Day 116: recurrent right pneumothorax due to cyst rupture in the right upper lobe (coronal section). **L** Day 116: recurrent right pneumothorax along with amelioration of the cystic lesion in the left lower lobe

An 81-year-old male, a non-smoker, presented to our hospital in January 2023 during the Omicron wave of the COVID-19 pandemic in China. He had been experiencing fever, cough, and expectoration for 14 days, despite self-symptomatic treatment, and presented progressive weakness. Notably, he had no history of exertional dyspnea. His medical history included rheumatoid arthritis without lung involvement, and he had received two doses of Sinovac COVID-19 Vaccine. On physical examination, he had a temperature of 38.8 °C, pulse rate of 105 beats/min, respiratory rate of 18 breaths/min, blood pressure of 150/90 mmHg, and percutaneous oxygen saturation of 95% on room air at rest. Chest auscultation revealed clear lung sounds. A reverse transcription-polymerase chain reaction (RT-PCR) test confirmed severe acute respiratory syndrome coronavirus 2 (SARS-Cov-2) infection. Blood tests showed elevated C-reactive protein (CRP) level of 121.83 mg/L, white blood cell (WBC) count within the normal range of 6.9 × 10^9^ /L, low lymphocyte count of 0.95 × 10^9^ /L, elevated alanine transaminase (ALT) of 103 U/L and aspartate transaminase (AST) of 142 U/L, and lactate dehydrogenase (LDH) of 437 U/L. A chest CT scan on day 14 from symptom onset revealed bilateral patchy consolidation and ground-glass opacities (Fig. 1A), consistent with moderate COVID-19. Due to his age, underlying conditions, weakened state, and history of receiving methotrexate before the onset of fever, moxifloxacin was given besides prone breathing and optimal supportive care, maintaining oxygen saturation above 93% without supplemental oxygen. However, on day 19, he developed progressive chest tightness and dyspnea, along with mental symptoms. Blood gas analysis (with 3 L/min oxygen administration) showed a pH value of 7.47, partial pressure of carbon dioxide (PCO_2_) of 32 mmHg, and partial pressure of oxygen (PO_2_) of 74 mmHg [PaO_2_/FIO_2_ (P/F): 224 mmHg]. Blood tests revealed a CRP of 77.93 mg/L, WBC of 6.3 × 10^9^/L with a lymphocyte count of 0.96 × 10^9^ /L, ALT of 45 U/L, AST of 36 U/L, LDH of 440 U/L, and elevated D-dimer of 38.5 mg/L. Subsequent chest CT scanning performed on day 20 showed the progression of lung lesions, and a subpleural air bronchogram in the anterior segment of the right upper lobe (Fig. 1B). Treatment escalated to include dexamethasone therapy (5 mg/day for 6 days) as severe COVID-19 was diagnosed, and moxifloxacin was halted based on its potential mental side effects. Although no signs of pulmonary embolism were found based on chest CT and echocardiography, anticoagulant therapy with low molecular weight heparin was administrated, as the B ultrasound of bilateral lower limb veins demonstrated highly probably thrombosis in the left peroneal vein and intramuscular veins. On day 26, the CT scan was repeated due to unimproved dyspnea and oxygenation status, indicating significantly aggravated lung consolidation as well as subpleural cystic lesions (4.5 cm × 3.5 cm and 2.5 cm × 1.7 cm, respectively). The proximal bronchi also had a thickened wall and constricted lumen in the anterior segment of the right upper lobe (Fig. 1C). Post-infection organizing pneumonia was suspected, and treatment was modified to methylprednisolone (40 mg/day) for enhanced effectiveness. As the patient was too weak to expectorate sputum, and glucocorticoid therapy was continued, the empirical ceftriaxone was administered, which gradually improved the patient’s condition. On day 38, his dyspnea and lung consolidation were significantly alleviated following anti-inflammatory treatment with methylprednisolone (40 mg/day for 7 days, and gradually tapered), in addition to optimal supportive care, prone breathing, and ceftriaxone treatment. Unexpectedly, subpleural cystic lesions in the anterior segment of the right upper lobe remained mostly unaltered, presenting as a cavity-like lesion with an air-fluid level in the dorsal segment of the right lower lobe (Fig. 1D), and another cystic lesion in the posterior basal segment of left lower lobe (Fig. 1E). No pathogen was found after repeated sputum smear staining and cultures, as well as the detection of 1,3-β-d-glucan (G test) and galactomannan (GM test) in serum, tuberculous interferon release assay, and X-pert MTB/RIF assay. Bronchoscopy was not performed as the patient and his family refused it. This culminated on day 42, when the patient presented with suddenly worsened dyspnea and severe cough, accompanied by reduced breath sounds in the right lung upon auscultation. Chest CT scanning demonstrated the evolution of the cavity-like lesion into an enlarged cystic lesion (5.7 cm × 4.0 cm) within the dorsal right lung, alongside right pneumothorax and subcutaneous emphysema (Fig. 1F, G). The rapid use of chest tube drainage and oxygen therapy produced an amelioration of the right pneumothorax and gradual resolution of the subcutaneous emphysema on day 50 (Fig. 1H), after which the patient was discharged. On day 80, a follow-up CT scan showed a gradual resolution of lesions in both lungs, but the subpleural cystic lesions were still present but constricted, with emphysema or air trapping, and bronchiectasis (Fig. 1I). On day 116, mild exertional dyspnea was observed, and chest CT demonstrated a recurrent right pneumothorax due to cyst rupture in the right upper lobe (Fig. 1J, K), alongside the improvement of cystic lesions in bilateral lower lobes (Fig. 1J, L). By day 156, the patient reported satisfactory recovery, with mild discomfort after exercise. Informed consent was obtained for the publication of this case.

## Literature review

Until September 1^st^, 2023, a total of 88 articles were retrieved by searching PubMed with keywords such as “COVID-19, bulla, pneumothorax,” or “COVID-19, cyst, pneumothorax,” or “COVID-19, pneumatocele, pneumothorax.” Alongside our case, we summarized the clinical data of 22 COVID-19 patients with cystic lesions termed “bulla,” “cyst,” or “pneumatocele,” and secondary spontaneous pneumothorax unrelated to underlying lung disease or mechanical ventilation [2–20] (Table 1). The median age of the 23 patients was 49.7 ± 13.8 years, with 95.6% (22/23) being male. Most patients (15/23, 65.2%) had no significant past medical history. A total of 14 patients (60.8%) were diagnosed with severe COVID-19, and eight patients (34.7%) with moderate COVID-19. On average, lung cystic lesions were detected on day 26.4 ± 9.8 from symptom onset, involving both lungs in 11 cases, the right lung in seven cases, and the left lung in five cases; an air-fluid level in the cystic lesion was observed in 9 of 23 (39.1%) patients. Cystic lesions were generally large, often exceeding 4 cm in diameter (20/23, 86.9%). As most patients (19/23, 82.6%) were in convalescence and discharged at home, intrapulmonary cystic lesions and spontaneous pneumothorax were typically identified concurrently when patients presented with sudden-onset pneumothorax symptoms (19/23, 82.6%). Pneumothorax occurred on average on day 29.5 (ranging from 18–35), involving the right side in 11 (47.8%) cases, the left side in seven (30.4%) cases, and bilaterally in five (21.7%) cases. Chest tube drainage effectively alleviated pneumothorax in most patients (20/23, 86.9%). While cystic lesions tended to improve over time, their disappearance may require several months, and one case experienced recurrent spontaneous pneumothorax. SARS-Cov-2 detection results were available in only seven cases when pneumothorax occurred, with four cases negative and three cases positive. Ultimately, all patients recovered except for one case, in which the cystic lesion appeared on day seven from symptom onset.

**Table 1:** Clinical data of COVID-19 patients with spontaneous pneumothorax secondary to lung cystic lesions

**Series No. [reference No.]**	**Sex, age**	**Chronic underlying disease**	**COVID-19 severity, oxygen therapy**	Determinate date*, location of cystic lesions	**Maximum diameter, air-fluid level of cystic lesions**	Onset date*, location of pneumothorax	**Inpatient or outpatient when pneumothorax occurs**	**Treatment of pneumothorax**	**RT-PCR result of SARS-CoV-19 when pneumothorax occurs**	**Patient outcome**
1 [2]	Male, 42	Not mentioned	Not mentioned	Day 35, right lower lobe	Large size, with air-fluid level	Day 35, right side	Outpatient	Thoracoscopic surgery	negative	Improved
2 [3]	Male, 57	None	Severe, nasal cannula	Day 13, bilateral lungs	Not mentioned, with air-fluid level	Day 15, left side	Outpatient	Chest tube insertion	Not mentioned	Improved, cysts left in the lung on day 63
3 [4]	Male, 60	Diabetes Mellitus	Severe, oxygen mask	Day 30, left lower lobe	15.2 cm, without air-fluid level	Day 30, left side	Outpatient	Chest tube insertion, left thoracotomy	Not mentioned	Improved
4 [4]	Female, 33	Obesity, Diabetes Mellitus	Severe, oxygen mask	Day 33, bilateral lungs	Large size, without air-fluid level	Day 33, right side	Outpatient	Chest tube insertion	Not mentioned	Improved
5 [5]	Male, 37	None	Moderate, no oxygen therapy	Day 30, bilateral lungs	Large size, with air-fluid level	Day 30, right side	Outpatient	Chest tube insertion	Positive	Improved, cysts left in the lung on day 60
6 [6]	Male, 54	None	Severe, high-flow nasal cannula	Day 18, left lung	Large size, without air-fluid level	Day 18, bilateral lungs	Outpatient	Chest tube insertion	Not mentioned	Improved
7 [7]	Male, 45	Hypertension	Moderate,no oxygen therapy	Day 35, bilateral lungs	6 cm, without air-fluid level	Day 35, bilateral lungs	Outpatient	Chest tube insertion	Not mentioned	Improved, cyst left in the lung on day 77
8 [8]	Male, 52	None	Moderate,no oxygen therapy	Day 16, right lower lung	No mentioned, without air-fluid level	Day 16, right side	Outpatient	Chest tube insertion	Not mentioned	Improved
9 [8]	Male, 63	Diabetes Mellitus, Hypertension	Severe, nasal cannula	Day 29, bilateral lungs	Large size, without air-fluid level	Day 29, left side	Outpatient	Chest tube insertion, thoracoscopic bullectomy and pleurodesis	Not mentioned	Improved, cyst left in the lung on day 60
10 [9]	Male, 38	None	Severe, nasal cannula	Day 14, right upper lung	4 cm, without air-fluid level	Day 14, right side	Inpatient	Conservative treatment	Not mentioned	Improved
11 [10]	Male, 41	None	Severe, nasal cannula	Day 40, left upper lobe	2.8 cm, without air-fluid level	Day 40, left side	Outpatient	Chest tube insertion	Negative	Improved lung cyst disappeared on day 135
12 [11]	Male, 77	None	Severe, nasal cannula	Day 21, right lower lobe	Large size, with air-fluid level	Day 19, right side	Inpatient	Chest tube insertion	Negative	Improved
13 [12]	Male, 32	None	Moderate,no oxygen therapy	Day 21, left lung	Large size, without air-fluid level	Day 21, left side	Inpatient	Chest tube insertion	Not mentioned	Improved, lung cyst disappeared on day 92
14 [13]	Male, 37	None	Severe, high-flow nasal cannula	Day 26, right lung	5.6 cm, without air-fluid level	Day 26, right side	Outpatient	Chest tube insertion	Not mentioned	Improved
15 [14]	Male, 63	None	Severe, not mentioned	Day 48, left lung	Large size, without air-fluid level	Day 48, left side	Outpatient	Chest tube insertion	Not mentioned	Improved
16 [15]	Male, 55	No mentioned	Moderate, no oxygen therapy	Day 30, bilateral lungs	Large size, with air-fluid level	Day 30, right side	Outpatient	Chest tube insertion, and thoracoscopic surgery	Not mentioned	Improved
17 [16]	Male, 40	None	Moderate,no oxygen therapy	Day 18, right lung	7.3 cm, with air-fluid level	Day 18, right side	Outpatient	Chest tube insertion	Not mentioned	Improved
18 [17]	Male, 68	None	Severe, face mask	Day 40, bilateral lungs	12 cm, without air-fluid level	Day 40, bilateral sides	Outpatient	Chest tube insertion	Not mentioned	Improved
19 [17]	Male, 40	None	Severe, mask	Day 7, right lung	Large size, without air-fluid level	Day 7, right side	Outpatient	Chest tube insertion	Positive	Died on Day 67
20 [18]	Male, 45	None	Moderate,no oxygen therapy	Day 26, bilateral lung	Large size, with air-fluid level	Day17, bilateral sides	Outpatient	Chest tube insertion	Not mentioned	Improved
21 [19]	Male, 46	hypertension	Moderate, no oxygen therapy	Day 21, bilateral lungs	Large size, with air-fluid level	Day 21, bilateral	Outpatient	Chest tube insertion	Not mentioned	Improved, cysts left in the lung on day 25
22 [20]	Male, 38	Not mentioned	Severe, oxygen mask	Day 32, bilateral lungs	5 cm, without air-fluid level	Day 32, left side	Outpatient	Conservative treatment	negative	Improved, cysts left in the lung on day 37
23^[this study]^	Male, 81	Rheumatoid arthritis	Severe, nasal cannula	Day 26, bilateral lungs	5.7 cm, with air-fluid level	Day 42, right side; Day 116, right side	Inpatient (first-time pneumothorax) and outpatient (second-time pneumothorax)	Chest tube insertion (first-time pneumothorax); Conservative treatment (second-time pneumothorax)	Positive (first-time pneumothorax)	Improved, constricted cysts were still present on day 116

## Discussion

Secondary spontaneous pneumothorax due to a lung cystic lesion termed “bulla” or “pneumatocele” is a rare complication in COVID-19 patients without underlying lung disease or mechanical ventilation, just as shown in our case. Until now, its prevalence, pathogenesis, risk factors, clinical course, and prognosis remain unclear. This report demonstrates the evolution of lung cystic lesions over four months and recurrent spontaneous pneumothorax due to the cystic lesions even three months after COVID-19 onset in a convalescent COVID-19 case. Based on the clinical data reviewed and summarized in this report from 23 patients, a chest CT examination may be necessary to determine if cystic lesions develop 2 to 3 weeks after symptom onset, especially in males with COVID-19 pneumonia. Cystic lesions should be monitored over several months or longer to be aware of spontaneous pneumothorax.

The prevalence of pneumothorax, possibly related to underlying lung disease or mechanical ventilation, is approximately 1% [19] in COVID-19 patients. Notably, it can occur in COVID-19 cases without ventilation procedures and a history of pulmonary disease, secondary to a new-onset cystic lesion of the lung. While the prevalence of spontaneous pneumothorax secondary to cystic lesions in COVID-19 patients is unknown, male gender appears to be prone to developing cystic lesions, and cysts with a large diameter (≥ 4 cm) seem to be a risk factor for spontaneous pneumothorax.

The pathogenesis of lung cystic lesions in COVID-19 patients remains unclear. Histological results suggest possible mechanisms involving diffuse alveolar damage, check-valve formation in the bronchus [19, 20], direct virus-induced airway injury via the angiotensin-converting enzyme (ACE) II receptor [21], and traction of fibrous tissue around the cyst [22]. In this report, cystic lesions occurred at the distal end of constricted bronchi at the right upper lobe, providing further support for this hypothesis. Increased intrapulmonary pressure, such as during coughing, can lead to cyst wall rupture and secondary pneumothorax.

In this report, a cystic lesion with a thickened wall and air-fluid level may resemble a “cavity” on CT images, but rapid evolution into a thin-wall cyst supports it as an uncommon COVID-19 finding termed pneumo-hamatocele (PHC). PHC, preferentially localized lung bases [23], results from a hematic accumulation due to micro-capillary bleeding with a secondary inflammatory fibrotic process. Along with blood reabsorption, the remaining lesion manifesting as a “giant bulla” is the “capsule” of inflammation, the thin wall of which is prone to rupture and result in pneumothorax [24]. Pneumothorax was reported in 81.1% (30/37) of COVID-19 patients with PHC, especially those with larger cyst diameters (≥ 5 cm) [24].

The first-time pneumothorax in our patient likely resulted from PHC rupture in the right lower lobe, while the second-time pneumothorax was due to cyst rupture in the right upper lobe. The corticosteroid therapy may contribute to the development of pneumothorax, but the persistent weakness of the involved lung tissue should be more important for the pneumothorax development, since the corticosteroid therapy had been stopped for more than 50 days when the second pneumothorax occurred. Although multifactorial, the formation of cysts and secondary pneumothorax primarily stems from enhanced lung inflammation. Whether early treatment with adequate steroids can prevent or accelerate the resolution of cystic lesions and spontaneous pneumothorax warrants further investigation.

The presence of pulmonary bullae has been associated with poor outcomes in COVID-19 patients with pneumothorax [25]. However, our results show that in convalescent COVID-19 patients with lung cystic lesions, the clinical outcome is satisfactory if the pneumothorax is appropriately diagnosed and treated. Conversely, pneumothorax occurring during the acute stage of COVID-19 is often fatal, likely due to the more severe COVID-19 infection and poor lung compensatory function. Similarly, the development of cystic lesions and secondary pneumothorax can be fatal for patients with underlying lung disease or those on mechanical ventilation.

## Conclusions

Recurrent spontaneous pneumothorax secondary to lung cystic lesions can occur in convalescent COVID-19 patients, leading to sudden respiratory function deterioration. Male individuals with COVID-19 pneumonia are particularly at risk and performing a chest CT examination 2 to 3 weeks after symptom onset may be necessary to identify the development of cystic lesions. These lesions may persist even after several months and potentially result in recurrent spontaneous pneumothorax. Apart from lung fibrosis, other lung sequelae such as cystic lesions, bronchiectasis [26], emphysema [27] or air trapping [28], as observed in our patient, may significantly impact the quality of life in convalescent COVID-19 patients. Hence, these sequelae warrant further attention and investigation in the future.

## Data Availability

All data and materials are provided in the manuscript.

## Abbreviations

COVID-19: Novel coronavirus disease 2019
PHC: Pneumo-hamatocele
CT: Computed tomography
RT-PCR: Reverse transcription-polymerase chain reaction
SARS-Cov-2: Severe acute respiratory syndrome coronavirus 2
CRP: C-reactive protein
WBC: White blood cell
ALT: Alanine transaminase
AST: Aspartate transaminase
LDH: Lactate dehydrogenase
PaCO2: Partial pressure of carbon dioxide
PaO2: Partial pressure of oxygen
ACE: Angiotensin-converting enzyme
